# How can the perceptions and experiences of medical educator stakeholders inform selection into medicine? An interpretative phenomenological pilot study

**DOI:** 10.15694/mep.2018.0000282.1

**Published:** 2018-12-11

**Authors:** Marise Lombard, Arthur Poropat, Louise Alldridge, Gary D. Rogers

**Affiliations:** 1 Griffith University; 2 Plymouth University

**Keywords:** Medical student selection, stakeholders, medical educators, phenomenology, interpretative phenomenological analysis.

## Abstract

This article was migrated. The article was marked as recommended.

Background

Attempts to utilise the experiences of stakeholders to better inform selection into medicine are rare in the literature. Published scholarship to date reflects a myriad of competing goals for selecting and graduating ‘good doctors’ amidst increasingly complex health care environments. This includes debates around what is the ‘good doctor’, selection methods, health care decision-making, the doctor-patient relationship, patient-centredness, professionalism and stakeholder experiences with doctors. Within the complexity manifested by these multiple dimensions, decisions about the characteristics and capabilities on which selection should be based may have privileged some stakeholder groups over others, with patient experiences particularly de-emphasised. The aims of this pilot study were to focus on front-line medical educators as stakeholders whose experiences might be valuable for informing selection into medicine and to inform a larger-scale study of the topic from the perspectives of a more diverse group of stakeholders, including patients.

Method

Fourteen (14) medical educator participants were recruited for a semi-structured group interview at an international conference for health professional educators. The audio-recording was transcribed verbatim and the raw data were de-identified and organised with the aid of computer assisted data analysis software. Coding was initiated and Smith’s interpretative phenomenological analytical (IPA) method employed ( Smith, Flowers, & Larkin, 2009).

Results

Initial analysis yielded four broad phenomenological themes: perceptions of ‘good doctors’, selection processes, selection-related challenges and possible solutions. The more deeply experiential data were captured in an analytical commentary of first-person accounts that may be useful for informing future selection strategies. Participant experiences mirrored the major debates in medical selection but their accounts revealed a negativity and cynicism about the topic that was concerning and warrants further investigation.

Conclusion

This study contributes to medical student selection research through offering an account of the ‘lived experiences’ of front-line medical educator stakeholders.

## Introduction

The desire to improve medical student selection has prompted researchers in the fields of science, philosophy, the social sciences, medical ethics and medical education to engage in a debate over many years ( Allen, Brown, & Hughes, 1997; Australian Medical Students Association, 2009; Benbassat & Baumal, 2007; Bore, Munro, & Powis, 2009; Burch, 2009; Caelleigh, 1990). Much of the research informing medical student selection has focused on how the selection process relates to the performance of candidates. Apart from trends that are impacting on medical education in general and on entry selection processes in particular, medical schools share challenges on a global scale. Examples include increased public scrutiny that demands greater accountability from health care professionals, sweeping changes in health care delivery and a fiercely competitive financial climate ( Aretz, 2011; Australian Health Practitioner Regulation Agency, 2011 ; Australian Medical Students Association, 2009; Marais, 2011; Mckimm & McLean, 2011; Pesce, 2010).

Universities remain under intense pressure to select from large applicant pools those candidates who can provide good returns on a community’s investment. Political and social factors also affect how medical schools select and educate their students. For instance, in Australia, planned health reforms have challenged medical schools to be more accountable for graduating a skilled medical workforce that meets the changing health needs of an increasingly diverse and ageing population ( Pesce, 2010; Roxon, 2010; Rudd, 2010; Trounson, 2011).

Accordingly, there is increasing interest in the contribution that the perspectives of stakeholders may add to the field of medical student selection. For the purpose of this paper we drew on the following two definitions of ‘stakeholder’:

Any party - person, group, or community - with an interest in a particular effect or outcome.

( Segen’s Medical Dictionary, 2012)

One who is involved in or affected by a course of action.

(Merriam-Webster Dictionary, 2017)

Examples of stakeholder informed selections scholarship include educator role-modelling ( Butani, Paterniti, Tancredi, & Li, 2013), cultural competency training ( Kamaka, 2010 ) and social accountability ( Preston, Larkins, Taylor, & Judd, 2016). More recently Kelly et al. (2018) published a systematic review of stakeholder views of medical selection methods.

Stakeholder-based research that has contributed to informing medical student selection and education includes multiple dimensions, the key ones of which are outlined as follows:

### Stakeholder perspectives of the ‘good doctor’

Attempts to inform medical selection and education from stakeholder perspectives of the ‘good doctor’ revealed studies that were limited in scope as well as in number. Of note was the predominance of quantitative research approaches ( Çetin et al., 2011; Fones, Heok, & Gan, 1998; Hurwitz et al., 2013; Lambe & Bristow, 2010; Linke, Chalmers, & Ashton, 1981) and of thematic-type analyses for most of the qualitative-based research ( Cuesta-Briand, Auret, Johnson, & Playford, 2014; Maudsley, Williams, & Taylor, 2007; Walsh, Arnold, Pickwell-Smith, & Summers, 2016). It is noteworthy that only two of the studies cited above focused on patients ( Fones et al., 1998; Walsh et al., 2016).

### Stakeholder views of selection methods

Although some of the research Kelly has conducted ( 2015) and led ( Kelly, Dowell, et al., 2014; Kelly, Gallagher, Dunne, & Murphy, 2014; Kelly et al., 2013) has involved quantitatively-based attempts to assess the effectiveness of selection tools (such as the HPAT-Ireland and MMI), the mixed methods study that she undertook for her doctoral research (2015) and her recent first-author publication ( Kelly et al., 2018) are important for informing medical student selection from a stakeholder perspective. Particularly relevant is Kelly’s qualitative approach to explore stakeholder acceptability of admissions in three medical schools in Ireland as part of her mixed methods study (2015). Kelly and colleagues (2018) underlined the importance of stakeholder perspectives in medical selection when they stated that:

[i]t is critical to the operation of fair and defensible selection processes that we understand and appreciate the range and depth of views that they [stakeholders] hold .. it highlights the need for better standards and more appropriate methodologies; for broadening the scope of the stakeholder groups included in future research. (p. 23)

### Stakeholders in health care

The role of stakeholders in health care decision-making is widely recognised in the literature. In the context of medical selection decision-making, such recognition includes stakeholder viewpoints related to medical workforce issues in health care delivery planning ( Hudson, Weston, & Farmer, 2017; Kecskes & Mitchell, 2017; Murray & Wilson, 2017; Patterson, Ferguson, & Knight, 2014; Tiffin, 2017). Health-care decision-making that is driven by patients is recognised more by other health care professions - nursing in particular - than by medicine ( Gillespie, Florin, & Gillam, 2004; Hook, 2006; Huffman, 2005). However, even in medicine there is consensus over the importance of patients’ safety and their health care rights, with some scholars focusing on patient satisfaction as a ‘core skill for medical students’ ( Robichaud, East, Beard, & Morra, 2012, p. 256). Further evidence to support the relatively poor recognition of patient perspectives in medical selection decision-making is found in research focused more on performance outcomes for the student/graduate/doctor than on patient satisfaction ( Bombeke et al., 2010; Brock, 2011; Gillespie et al., 2004; Luxford, 2011). These scholars support criticism of a medical student selection process that has ‘privileged’ the interests of some stakeholders above others ( Lombard, Rogers, Poropat, & Alldridge, 2013, 2017; MacLeod, 2011; Young, 2010).

### The doctor-patient relationship

Broadly speaking, discourses around the doctor-patient relationship explore the tension between the ‘art’ (humanism) and the ‘science’ (knowledge) of medicine as well as the ‘good doctor’ phenomenon from multiple stakeholder perspectives. For many scholars empathy is claimed to be at the centre of the doctor-patient relationship, with studies by Hojat and colleagues leading the field ( Hojat, 2007, 2014; Hojat, Axelrod, Spandorfer, & Mangione, 2013; Hojat, Mangione, Kane, & Gonnella, 2005; Hojat et al., 2004; Hojat et al., 2015; Hojat et al., 2009; Hojat & Zuckerman, 2008; Hojat et al., 2005). Interesting to note is the wide recognition given to claims of progressive declines in students’ empathy during medical programs. Although there is research evidence to support such claims ( Chen, Lew, Hershman, & Orlander, 2007; Neumann et al., 2011; Pedersen, 2009), more recent evidence has brought them into question ( Bombeke, De Winter, & Van Royen, 2014; Colliver, Conlee, Verhulst, & Dorsey, 2010; Ferreira-Valente et al., 2017; Roff, 2015). Added dimensions relate to scholars who contextualise empathy synonymously with altruism ( Burks & Kobus, 2012; Jones, 2002) or with compassion ( Cameron et al., 2013; Carmel & Glick, 1996; Green, 2013; Lown, Rosen, & Marttila, 2011). Closely aligned with discourses around empathy, altruism and caring, is research focused on the importance of emotional intelligence (EI) for healthy doctor-patient relationships ( Johnson, 2015; Weng et al., 2008). Aside from empathy and EI, communication is widely recognised as a key element of the doctor-patient relationship ( Abadel & Hattab, 2014; Best, 2011; Casey et al., 2014; Kelly et al., 2013; Lim et al., 2012; Turner et al., 2016). A study by Maudsley et al. (2007) focused on medical students as stakeholders who had shared their perspectives and expectations of the ‘good doctor’. These scholars proposed that further evidence was needed to ‘support ongoing commentary about patients seeking qualities related to communication, caring, and competence in doctors’ (p. 476).

### Patient-centredness

This dimension was strongly linked to that of the doctor-patient relationship in stakeholder-based research that informs medical selection decision-making. Referred to in some studies as patient-centred care (PCC), this dimension is also linked to discourses around the ‘hidden curriculum’ in medical education. For example Bombeke et al. (2010) and Haidet (2010) promoted PCC from a stakeholder perspective that was student-centred and that supported medical educators and students to develop PCC in practice. Haidet (2010) contended that:

patient-centredness is difficult to achieve in practice, because it challenges prevailing professional norms .. it is difficult to see the points at which the patient’s perspective is at odds with our own, to respect such differences, and to give the patient’s perspective as much weight as our own while trying to reconcile differences between the two. (pp. 643-644)

An Australian-based study by McNair, Griffiths, Reid and Sloan (2016) applied a theoretical framework for patient-centredness from patient and doctor perspectives, whilst, according to Little et al. (2001) a patient-centred approach includes communication, partnership and health promotion for vulnerable patients. Coelho (2010) proposed a policy framework for ‘patient-centred comparative effectiveness research’ that was noteworthy for including multiple stakeholders, especially patients. Mainous, Goodwin and Stange (2004) analysed data from 138 family physicians (GPs) and 4,454 patients to explore shared perceptions and to advocate for increased continuity of care for patients. Although the authors used a quantitative approach, their large-scale study was notable and made a valuable contribution to stakeholder-based discourses around patient-centredness.

These studies provide a rich background for ongoing work to ensure that the perspective of stakeholders informs medical selection practice, but accounts of the experiences of front-line medical educator appear to be largely absent from the literature.

## Methods

An opportunity arose to undertake a pilot study when the first author was invited to lead a 45-minute ‘Personally-Arranged Learning Session’ (PeArLS) at an international health professional educators’ conference in Australia ( Lombard et al., 2011). The conference theme was ‘ethics and education’ and the abstract was titled: *What makes a good doctor? How would patients like us to select medical students?* Ethics approval was granted by Griffith University’s Human Research Ethics Committee (Reference number med/21/11/hrec). Additional approval was granted by conference organisers to conduct the session as a discursive group interview, including audio-recording.

The aim of the group interview was to explore how participant perceptions and experiences could better inform selection into medicine. Of the 27 conference delegates who attended the PeArLS, 14 consented to be audio-recorded for the group interview. Excluding one medical selections officer and one medical student, the remaining twelve participants identified as medical educators from medical schools in Australia and abroad and were thus recruited for this study for their interest and expertise in the topic. They shared academic and clinical backgrounds across several health professions that included medicine, nursing, physiotherapy and occupational therapy. The setting was a conference room and the group interview was conducted for the 45-minutes allocated to the PeArLS. Participant consent was confirmed after reading the interview information handout. Notetaking was undertaken by a confederate during the interview. The audio-recording was transcribed verbatim in conjunction with note-checking and discussion with senior colleagues post-interview. After de-identification, the transcript was read and re-read to identify patterns and emerging themes and coding was undertaken with the aid of computer assisted data analysis software.

Phenomenology offered the most suitable paradigm for exploring participant experiences in relation to the ‘good doctor’ phenomenon in ways that were useful for informing selection into medicine. As the analysis progressed, Smith’s IPA method ( 2009) was applied to explore participant experiences more deeply in a hermeneutic cycle of reciprocal meaning-making and interpretation.

## Results/Analysis

From a total of 14 participants in the semi-structured group interview, the data analysed in this paper were collected from the twelve consenting medical educators. The conference session titled *What makes a good doctor? How would patients like us to select medical students?* contextualised the prompt questions. Participant responses were anonymised and allocated numbers 1.1 to 1.12.

The results of the data analysis are summarised in Table 1. The first column delineates the four phenomenological themes that emerged around the ‘good doctor’, selection methods, selection challenges and possible solutions. The second column ( Table 1) identifies smaller units of meaning that emerged progressively as the data were coded to reveal deeper themes. The third column ( Table 1) provides illustrative quotations selected to reflect each unit of meaning and theme in greater depth.

**Table 1.** Data Analysis Structure

Phenomenological theme	Unit of meaning	Illustrative quotations
**1. ‘Good doctors’**	1.1 Effective interpersonal skills	*I think the biggest one is interpersonal skills, so basically communication that is easy, comfortable ... I want them* [doctors] *to be professional but I don’t want to have an alienating experience ... I certainly want to be listened to as a patient, it’s my body ...* [Participant 1.3].
	1.2 Empathic	*My most memorable experience as a patient was ... I had clinical depression and I went to a GP that was recommended by a friend of mine ‘cos she was really good. I told her about what was happening with my life ... she was really empathic, saying things like ‘Ah your life really sucks, it must be really hard; I don’t know how you do that’* [Participant 1.7].
	1.3 Doctors-in-training	*We have very ‘bad’ students with very high grades and we’ve got ‘fabulous’ students with low grades. At our* [Australian-based] *medical school the assumption that students with good grades make ‘good doctors’ has not been the case* [Participant 1.5].
**2. Medical student selection methods**	2.1 Academic scores	*Across the board* [names an Australian university] *has decided academic results ... full stop that’s it. And the cut off is until the course is full. And it hasn’t made a blind bit of difference ... but you may just as well take anyone and see what happens really!* [laughter from the group] [Participant 1.5].
	2.2 Combined aptitude and metric tests	*I would say to date the various ‘SATS’* [referring to the UMAT and GAMSAT] *have not been successful* [Participant 1.4].
	2.3 Traditional interviews	*We do* [traditional] *interviews, which are **incredibly** labour-intensive and expensive! There’s a high likelihood that we’re getting rote answers even ‘though we write new vignettes every two to three years. We have to acknowledge that* [traditional] *interviews are a flawed process as we still get a handful of what you could call ‘sociopaths’* [Participant 1.3].
	2.4 Multiple mini interviews	*We’ve just changed our admission procedure ...* *We used to do a* [traditional] *interview process, the problem being everyone knew what our interview questions were! We’re going to do multiple mini interviews that will involve the community as well as doctors ... it’s kinda the best that you can do* [Participant 1.12].
	2.5 Psychometric testing	*We’re doing is a PQA* [personal qualities assessment], *which is a personality questionnaire that excludes on the extremes of personality over five criteria so that we don’t get the few people with ‘personality disorders’ that take up 90 per cent of our time* [Participant 1.11].
**3. Medical student selection challenges**	3.1 School-leaver candidates	*How can we be assessing those kinds of characteristics that are going to determine a ‘good’ doctor in young people with no life experience ... trying to select who’s going to be a ‘good’ doctor is a bit of a ‘furphy’* [untruth] *! How can you determine the resilience of 18 and 19-year-olds?* [Participant 1.9].
	3.2 Reliability and validity of selection tools	*I’ve been through training traditional* [undergraduate] *students, graduate-entry students, students of all ages and from all sorts of backgrounds and from my experience selection has made no difference* [Participant 1.7].
	3.3 Conflicting interests	*I don’t think the* [traditional] *interview really helps to weed out the really, **really** ‘bad’ ones who lie, steal, cheat and do really terrible things! We then have a lot of trouble getting anybody, including the medical board or the university to take matters seriously. There are also inconsistencies like they* [medical students] *need police checks to get into clinical placements, yet a police check is not a pre-entry requirement at our* [Australian] *medical school* [Participant 1.6].
	3.4 Selection error	*I would say every cohort ... we get between four and six* [medical] *students who are ‘criminal’, ‘unethical’, ‘amoral’ ... who really just should not be there and once they are there we have a major problem. I don’t think selection has helped us very much to weed out that sort of person or the ones who don’t really want to be there* [Participant 1.6].
**4. Medical student selection solutions**	4.1 Increase diversity	*We need to select* [medical] *students for the places that we’re servicing. We have a responsibility to produce graduates who are equipped to practice in a variety of places, we don’t want to pigeon-hole them* [Participant 1.3].
	4.2 Determine capabilities assessable on admission	*There’s always going be some people that slip through the selection process no matter what you do. We have tried to focus our multiple mini interviews on people who will be good medical students rather than doctors because we realise it’s our job to turn them into good doctors* [Participant 1.1].
	4.3 Determine capabilities that can be learned	*We have a strong responsibility to pick up those professional issues through the course and weed them out before they’re interns. We’re trying to make professional behaviour assessable and what is assessed for is a hurdle. I think it’s scary and we’re all frightened of pulling people up but we’ve all seen it in the system ... it’s got to be something the university will accept for rules of exclusion. That’s why we’ve made it into assessment because beforehand we couldn’t get rid of them and they did take up 90 per cent of our time and then they’re interns and then God knows what happens to them* [Participant 1.12].
	4.4 Anticipate hidden curricular effects	*The first years express some very idealistic notions ... I see it in years three and four that they do start to get ‘squashed’ by what we call the ‘hidden curriculum’ ... so perhaps we need to select people that are socially robust and resilient that they can withstand the ‘hidden curriculum effect’* [Participant 1.8].

## Discussion

In response to the first part of the pilot study research question, capabilities identified in the experiences of first-line medical educators in relation to the ‘good doctor’ included effective interpersonal skills and empathy in healthy doctor-patient relationships, which mirrored the literature. The second part of the pilot study research question, focused on selection into medicine, elicited participant responses relating to selection processes, challenges and solutions that were similarly evidenced in the literature. Useful for informing future selection into medicine was the emphasis placed by medical educators on the challenges facing medical schools. They raised specific issues relating to school-leaver candidates, to the reliability and validity of selection methods, to conflicting interests between medical schools, university boards, clinical environments and health care consumers, as well as to the consequences of selection error. The solutions they proffered were equally valuable for informing future selection into medicine. They urged medical schools to increase the diversity of candidates in a climate of social accountability, as well as to determine which capabilities for long-term, effective medical practice were assessable on admission and which could be acquired by graduation and beyond. They emphasised the need to address hidden curricula effects, particularly those that impacted negatively on trainee doctors and consequently on their patients.

Although the issues identified by this study are not new to the medical student selection debate, what sets this study apart is the novel way in which the experiences of participants was captured to relay powerful messages for better informing selection into medicine. Equally noteworthy was the negativity and cynicism shared by these medical educator stakeholders in their attempts to identify and to address medical selection related challenges. The analytical commentary that illustrates how these shared patterns of negativity and cynicism emerged is too lengthy and detailed for this paper. However, the following first-person account goes some way to illustrating this phenomenon:

I think the selection process is inherently flawed .. totally! What we have to remember is we’re selecting medical students .. not practitioners .. I think the harsh reality is that the selections process is to weed down from your 1500 applicants to your 150 places .. we talk all the niceties around it but I think that’s the harsh reality. [Participant 1.4]

The research undertaken by Kelly et al. (2018) enabled useful comparisons between preferences expressed by some of the pilot study participants and those identified for stakeholders in the 71 studies she reviewed. The authors in Kelly’s review ( 2018) cited support for interviews, particularly MMIs (p. 18) and mixed responses to aptitude tests (p. 19), which concurred with our pilot study analysis. However, there is a noteworthy distinction that needs to be drawn between the Kelly-led attempts to better inform medical student selection ( 2013, 2014a, 2014b, 2015, 2018) and this study. While these authors linked stakeholder views to medical selection tools, our IPA approach focused on experiences of front-line medical educator stakeholders in ways not previously evidenced in the literature.

In addition to the study’s strengths, it is important to acknowledge its limitations. We measured our study against four qualitative criteria in the Guba and Lincoln (1989) framework as follows:

**Credibility**,which establishes ‘a match between the constructed realities of respondents (or stakeholders) and those realities as represented by the evaluator and attributed to various stakeholders’ ( Guba & Lincoln, 1989, p. 237). Attempts were made to address issues around analytical subjectivity to give credibility to the study. Strategies included debriefing with peers and supervisors; ‘bracketing’ or putting aside personal views and experiences; and being ‘reflexive’ by acknowledging and examining the potential influence of the interviewer’s role on participants’ responses.

**Transferability**, defined as the extent to which a reader is justified in applying the findings to their own circumstance. According to Guba and Lincoln (1989) ‘[t]he major technique for establishing the degree of transferability is thick description’ (p. 241). Although the single group interview chosen for the study limited the yield of sufficiently thick and rich data, there were repeated examples of issues being raised that were subsequently discussed in depth and extended in coverage, encouraging the further use of this method. Further, the findings can readily be applied to different settings with other ‘respondents’ ( Frambach, van der Vleuten, & Durning, 2013; Korstjens & Moser, 2017). The study was, therefore, useful for informing larger-scale, stakeholder-based research by demonstrating the need to explore the ‘good doctor’ phenomenon in greater depth with an increased diversity of participants, while attending to effective and broad recruitment and encouraging discussion and development of ideas.

**Dependability**, defined as the extent to which the findings may be relied upon and might be expected to be replicated if the study were repeated in the same way. Guba and Lincoln (1989) suggest that ‘[t]he technique for documenting the logic of process and method decisions is the dependability audit’ (p. 242). The study met this requirement by outlining research steps to address the research question, by referring to the literature and by remaining close to the data. The limited time meant data saturation was not achieved after the single group interview and participants did not evaluate the findings. Nonetheless, it was possible to verify that the data matched the apparent ‘saturation’ of the existing literature ( Frambach et al., 2013; Korstjens & Moser, 2017), with a similar range of topics raised.

**Confirmability**, defined as the extent to which the findings might be confirmed by other researchers utilising the same techniques with similar participants. Attempts to achieve confirmability of study data included keeping a diary, compiling field notes, referring to the literature, peer debriefing and data checking between authors. We tried to minimise bias by being transparent, by ‘bracketing’ data during analysis and through critical self-reflection ( Frambach et al., 2013; Korstjens & Moser, 2017).

## Conclusion

Overall, the aim of the study of informing selection for entry into medicine through examination of the lived experience of front-line medical educators was achieved. Although the study found that medical educator stakeholders’ perceptions of the ‘good doctor’ were mirrored in scholarly literature, their experiences of selection methods, of selection-related difficulties and of their attempts to overcome these were unique and somewhat negative and cynical. The study contributes to ongoing attempts to inform selection for entry into medicine and highlights the concerns of medical educator stakeholders. Further research is needed to explore stakeholder experiences with doctors and medical students in greater depth and from a more inclusive and diverse stakeholder perspective.

## Take Home Messages

• Employing a method based on interpretive phenomenological analysis is useful for exploring the lived experiences of stakeholders for better informing selection into medicine.
• A focus on stakeholder contributions to medical selections research has the potential to better inform medical student selection from more diverse and inclusive perspectives.

## Notes On Contributors

**Dr Lombard** (AFANZAHPE, M. Soc. Sci. (Nursing/Midwifery) RN, RM, IPN, NMCSP) lectures fulltime in Griffith University’s School of Medicine and has clinical and research interests in medical education, women’s health and community-based nursing and midwifery practice.

**Dr Poropat’s** (Bachelor of Arts, B Arts (Hons), M Org Psychology, PhD) work has focused on how personality and relationships guide learning and performance in education and work. His research has been published in *Psychological Bulletin* and *The Lancet*, and featured in *The New York Times* and *The Huffington Post.* ORCID number: https://orcid.org/0000-0002-8496-1815

**Associate Professor Alldridge** (BSc (Hons) Biology, M Learning and Teaching, PhD) is a fulltime senior academic tutor and the lead for the discipline of cell and molecular biology at Plymouth University. She is leading the design and implementation of a longitudinal, holistic widening participation strategy at Peninsula Medical School that aims to support students from disadvantaged backgrounds to access and succeed in medicine.

**Gary D. Rogers** MBBS(Adel.), MGPPsych(Monash), PhD(Adel.), FANZAHPE, FAMEE, PFHEA is Professor of Medical Education and Deputy Head of School (Learning & Teaching) at the Griffith University School of Medicine; Program Lead in Interprofessional Learning for the Griffith Health Institute for the Development of Education and Scholarship (Health IDEAS); and Senior Medical Officer, Gold Coast University Hospital. ORCID number: https://orcid.org/0000-0003-4655-0131
