# Supplementary material for: How can the perceptions and experiences of medical educator stakeholders inform selection into medicine? An interpretative phenomenological pilot study
Source: MedEdPublish (2016). 2018 Dec 11;7:282. [Version 1] doi: 10.15694/mep.2018.0000282.1 (PMC10711958; doi:10.15694/mep.2018.0000282.1)
Supplement: Peer review history

# Reviewer report: Reviewer response for version 1

**DOI:** 10.21956/mep.19726.r29545

**Published:** 2018-12-19

**Authors:** Jan Illing

**Affiliations:** 1 Newcastle University

This review has been migrated. The reviewer awarded 4 stars out of 5 This is an interesting paper and highlights many of the strengths and weakness of selection for medical school. The abstract and paper are clearly written, although I think the aim could be restated to be clearer and the two parts of the research question set out in the discussion should be presented up front. The introduction covers a range of relevant issues for medical selection and focused on some current themes of interest such as patient-centeredness and empathy. The methods used to set up the single group are adequately described, although the topic guide and details about how the analysis was conducted are not provided. The results in the table are the most interesting section and the quotes are real dynamite. However, they are presented in a table without comment or interpretation and briefly covered in the discussion. I would have preferred more discussion about these themes and how they inform the current research questions and those going forward; highlighting the strengths and weaknesses and informing future directions. This study has several limitations: self-selection, sample size, lack of data saturation most of which are covered in the paper however, I would have preferred a longer discussion of the findings and a shorter limitations sections.

Reviewer Expertise:

NA

No decision status is available

# Reviewer report: Reviewer response for version 1

**DOI:** 10.21956/mep.19726.r29544

**Published:** 2018-12-14

**Authors:** Richard Hays

**Affiliations:** 1 James Cook University

This review has been migrated. The reviewer awarded 4 stars out of 5 Thanks you for the invitation to review this paper. The topic is interesting and always topical, the content is familiar to me ( as an academic in medicine) and the issues identified in the thematic amanlysis come as little surprise. I have a few comments that may controvubute to some improvement. First, I am not sure that the authors are clear enough about the purpose of selection. I find this to be a big issue to discuss in interviewer training. Are we selecting to be a successful medical student or a great clinician? Second, I agree that this is a relatively shallow look at a wide range of issues. Nothing really new or different has emerged, perhaps because the analysis did not look deeper? Third, how important are the views of academics in medicineanyway? We are the implementer of policies developed by sometimes powerful stakeholders with strong views of what is required. I wonder if the value of interviews is mostly in face validity for certain stakeholders and to engage our communities. Both are good reasons, but we risk playing to an audience that believes good/bad applicants can be identified by human judgement (a little like viva voce examinations). I am not sure that medical academics should be stakeholders in medical student selection, as we will probably favour those who match our own biases. Finally, I am not sure that the alternatives to complex selection processes are dealt with well enough. At the centre is academic merit, which predicts the next academic performance. Should there be no attempt to assess personal qualities? Nevertheless, I enjoyed the paper, perhaps because I share many of the views. A deeper analysis and discussion would earn an extra star.

Reviewer Expertise:

NA

No decision status is available
